# Rationale for issuing neuroimaging requests for patients with primary headaches in China

**DOI:** 10.1002/brb3.3583

**Published:** 2024-06-06

**Authors:** X. M. Zhong, L. C. Zhao, L. L. Peng, L. Li, C. Q. Li

**Affiliations:** ^1^ Chengdu Second People's Hospital Chengdu China; ^2^ Department of Neurology The Second Affiliated Hospital of Chongqing Medical University Chongqing China

**Keywords:** decision‐making, head computed tomography, head magnetic resonance imaging, neuroimaging, primary headache

## Abstract

**Objective:**

To investigate the prevalence of neuroimaging in patients with primary headaches and the clinician‐based rationale for requesting neuroimaging in China.

**Data sources and study setting:**

This study included patients with primary headaches admitted to hospitals and clinicians in China. We identified whether neuroimaging was requested and the types of neuroimaging conducted.

**Study design:**

This was a cross‐sectional study, and convenience sampling was used to recruit patients with primary headaches. Clinicians were interviewed using a combination of personal in‐depth and topic‐selection group interviews to explore why doctors requested neuroimaging.

**Data collection:**

We searched for the diagnosis of primary headache in the outpatient and inpatient systems according to the International Classification of Diseases‐10 code of patients admitted to six hospitals in three provincial capitals by 2022.We selected three public and three private hospitals with neurology specialties that treated a corresponding number of patients.

**Principle findings:**

Among the 2263 patients recruited for this study, 1942 (89.75%) underwent neuroimaging. Of the patients, 1157 (51.13%) underwent magnetic resonance imaging (MRI), 246 (10.87%) underwent both head computed tomography (CT) and MRI, and 628 (27.75%) underwent CT. Fifteen of the 16 interviewed clinicians did not issue a neuroimaging request for patients with primary headaches. Furthermore, we found that doctors issued a neuroimaging request for patients with primary headaches mostly, to exclude the risk of misdiagnosis, reduce uncertainty, avoid medical disputes, meet patients’ medical needs, and complete hospital assessment indicators.

**Conclusions:**

For primary headaches, the probability of clinicians requesting neuroimaging was higher in China than in other countries. There is considerable room for improvement in determining appropriate strategies to reduce the use of low‐value care for doctors and patients.

## Graphical abstract

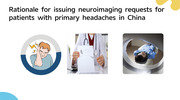

## 1 INTRODUCTION

Headache is an ancient problem that has plagued a large proportion of the population (Koehler & Boes, 2010; Rizzoli & Mullally, 2018). The lifetime prevalence of any type of headache is 96% (Jordan & Flanders, 2020). Currently, headache disorders rank third among global causes of disability, accounting for more than $78 billion per year of direct and indirect costs in the United States (Global Burden of Disease Study 2013 Collaborators, 2015; Mafi et al., 2015). Much criticism has been generated regarding the overuse of advanced imaging modalities for chronic headaches, given that most patients with a lack of focal neurological signs or symptoms have negative results (Cote & Laws, 2017; Heetderks‐Fong, 2019; Jordan & Flanders, 2020). A study conducted in China in 2018 examined 1070 healthy control patients and 1070 patients with primary headaches; imaging evaluation included either computed tomography (CT) or magnetic resonance imaging (MRI) and found no statistical difference in the detection of intracranial abnormalities: 0.58% in patients with headache and 0.78% in healthy controls (Wang et al., 2019). Similar studies have suggested that imaging is not cost‐effective in patients with chronic headaches without focal neurological signs or symptoms (Katzman, 1999; Morris et al., 2009; Vernooij et al., 2007). The Choosing Wisely Commission considers neuroimaging to be low‐value care for patients with primary headaches and advises against imaging (Cote & Laws, 2017; Wang et al., 2019). Low‐value medicine refers to medical practices that bring little or no benefit to patients and may cause varying degrees of physical, mental, and financial harm (Cote & Laws, 2017; Heetderks‐Fong, 2019).

China faces many challenges in meeting residents’ demands for medical and health services as their costs continue to increase. These include the abuse of antibiotics, excessive surgery, and unnecessary CT examinations, which not only do not benefit patients but may also cause harm, waste medical resources, and increase medical costs (Shang et al., 2019, 2016). Several studies have been conducted on the overuse of antibiotics and excessive surgery in China (Shang et al., 2019, 2016); however, few studies have been conducted on unnecessary CT scans. Therefore, this study aimed to investigate the prevalence of neuroimaging (CT/MRI) in China and determine clinician‐based rationales for issuing neuroimaging requests to patients with primary headaches.

## 2 METHODS

### 2.1 Neuroimaging investigation in patients with headaches

#### 2.1.1 Choice of hospital

We used an encounter sampling method to investigate neuroimaging findings among patients with headaches admitted to hospitals in three Chinese provincial capitals in 2022. We surveyed public and private hospitals using a random encounter sample to understand the differences between the business entities. Hospitals in China are either public or private based on ownership. Public hospitals are state‐owned (sponsored by government departments, state‐owned enterprises, and public institutions) and collectively owned. Private hospitals are hospitals other than public hospitals. The former are nonprofit hospitals, whereas the latter are mostly for‐profit hospitals. Hospitals in China practice classified management. Hospitals were divided into levels I, II, and III, according to their functions and tasks. Level I general hospitals are primary medical institutions that provide basic medical, preventive, health, and rehabilitation services to a community (population generally less than 100,000); level II hospitals serve areas containing multiple communities (population generally 1 million), and level III hospitals serve areas containing multiple districts (population generally more than 1 million). For this survey, we selected three prefecture‐level cities covering seven hospitals with large populations and high medical‐level accessibility. To understand whether the management body would lead to differences in the results, we selected three public and three private hospitals with neurology specialties that treated a corresponding number of patients.

#### 2.1.2 Collection of data

The study design was approved by the Medical and Health Research Ethics Committee of the Second People's Hospital of Chengdu, China. We searched for the diagnosis of primary headache in the outpatient and inpatient systems according to the International Classification of Diseases‐10 code (migraine, G43; cluster headache, G44.0; vascular headache, G44.1; tension‐type headache, G44.2; drug‐induced headache, G44.4). Specialist neurologists reviewed the patients’ cases. The inclusion criteria were age >12 years and diagnosis of primary headache at the first medical visit. Exclusion criteria were headache with any of the following “red flags”; sudden onset (“thunderclap”), features of intracranial hypertension or hypotension, new onset or pattern during pregnancy or peripartum period, increasing frequency or severity, fever or neurologic deficit, history of cancer or immunocompromise (e.g., human immunodeficiency virus infection), older age (>50 years) at onset, posttraumatic onset, headache‐causing awakening from sleep, or progressively worsening headaches. Information on patients who met the inclusion criteria was collected, including name, sex, age, route of visit (outpatient or inpatient), and type of completed neuroimaging (CT/MRI).

### 2.2 Clinician's interview

#### 2.2.1 Selection of clinicians

China's tertiary public hospitals implement strict three‐level diagnostic and treatment systems. Doctors in China are classified into four grades: (1) resident clinicians, (2) attending clinicians, (3) associate chief clinicians, and (4) chief clinicians. To avoid the influence of different hospitals and their clinicians, we interviewed clinicians with different titles from different hospitals. As clinicians with different professional titles vary in age, culture, and clinical experience, we selected four chief clinicians (two public and two private), four associate chief clinicians (two public and two private), four attending clinicians (two public and two private), and four residents (two public and two private).

#### 2.2.2 Personal in‐depth and topic group interviews

For personal in‐depth interviews, we required all clinicians to be in a quiet room; one interviewer was responsible for the interview, a recorder was responsible for recording and sorting out the recording, and we used a semi‐structured interview tool to ask questions from each interviewee (Table 1). Immediately after each interview, the recordings were transcribed by two investigators. We used a grounded theory approach to encode the text of each in‐depth interview to analyze the clinicians’ in‐depth perceptions of performing neuroimaging in patients with primary headaches. NVivo Qualitative Software was used to code the responses from resident clinicians, attending clinicians, associate chief clinicians, and chief clinicians and develop themes in each domain to generalize more realistic concepts. Four investigators read all coding results and met in person to discuss possible topics and differences in coding to find a consensus and to identify the final coding rules. After the personal in‐depth interviews, a topic selection group interview was conducted to further discuss the questions. Based on their titles, the clinicians were assigned to four topic groups. The interviewers in the topic selection group collectively scored them according to their reasons for personal in‐depth interview coding, summary, and analysis. They selected the most important items from all the answers listed, arranged them in order, and assigned each item a point according to its importance. The score was generally between 1 and 10 points, with the most important being 10 points and the least important being 1 point. The investigators calculated the scoring results for the group members and obtained a score value for each item. The investigators ranked the results on the basis of the scores. The first arrangement represented the common opinions of the group.

**TABLE 1:** Interview tool.

1. If you encounter a patient with primary headache through consultation and physical examination during outpatient and inpatient examination, will you advise neuroimaging?
A. Yes, and why?
B. No, and why?
2. Do you think neuroimaging is excessive or low‐value care for each patient presenting with primary headaches? (Low‐value care is defined as medical services that lack clinical benefit, have little benefit compared to cost, or have great potential harm (1,3,7,8))
A. If Yes: do you have any suggestions for reducing this phenomenon?
B. No, and why?

## 3 RESULTS

### 3.1 The neuroimaging rate for primary headache

This study investigated six hospitals in three provincial capitals, including three public and three private hospitals, involving 2263 patients. Of these, 232 (10.25%) patients did not complete the examination, including 209 outpatients and 23 inpatients. A total of 1942 patients (89.75%), including 822 outpatients and 1206 inpatients, completed the examination. Among them, 628 underwent head CT, 1157 underwent MRI, and 246 underwent both CT and MRI. The imaging rates in the three public hospitals ranged from 80.47% to 93.09%. The imaging rate of the three private hospitals was 90.40%–95.86% (Table 2).

**TABLE 2:** Primary headache neuroimaging.

City	Public or private	Total	Outpatient	Inpatient	Outpatient					Inpatient					Total	
					Not requested	Requested				Not requested	Requested				Not requested	Requested
						CT	MRI	CT + MRI	Total		CT	MRI	CT + MRI	Total		
A	Public	376	181	195	26 (14.36%)	100	52	3	155 (85.64%)	0	30	113	52	195 (100%)	26 (6.91%)	350 (93.09%)
	Private	319	136	183	29 (32.79%)	38	67	2	105 (77.21%)	0	0	182	1	183 (100%)	29 (9.09%)	290 (90.91%)
B	Public	444	181	263	29 (16.02%)	100	51	1	152 (83.98%)	4 (1.52%)	19	88	152	259 (98.48%)	33 (9.30%)	322 (90.70%)
	Private	362	156	206	15 (9.62%)	77	64	0	141 (90.38%)	0	3	198	5	206 (100%)	15 (4.14%)	347 (95.86%)
C	Public	466	253	213	77 (30.83%)	80	95	1	175 (69.17%)	14 (6.58%)	24	146	29	199 (93.42%)	91 (19.53%)	375 (80.47%)
	Private	296	127	169	33 (25.98%)	51	43	0	94 (74.02%)	5 (2.96%)	106	58	0	164 (97.04%)	38 (9.60%)	258 (90.40%)
Total		2263	1034	1229	209 (20.21%)	446	372	7	822 (79.79%)	23 (1.87%)	182	785	239	1206 (98.13%)	232 (10.25%)	1942 (89.75%)

Abbreviation: CT, computed tomography; MRI, magnetic resonance imaging.

### 3.2 Interview content

#### 3.2.1 Neuroimaging decisions and reasonings

We conducted in‐depth personal interviews with 16 clinicians, including four chief clinicians, four associate chief clinicians, four attending clinicians, and four resident clinicians. Six clinicians were interviewed online, and 10 were interviewed in person. Among the 16 interviewees, only one chief clinician stated that she would not perform neuroimaging for patients with primary headaches during consultations and physical examinations. Instead, she would perform a short‐term follow‐up observation, whereas other doctors at all levels said that they would perform CT or MR scans.

Based on the content of the personal in‐depth interviews, we found that clinicians would perform neuroimaging for patients with primary headaches to exclude the risk of misdiagnosis, reduce the uncertainty of diagnosis, avoid medical disputes, meet the medical needs of patients, and complete hospital assessment indicators, which refer to the link between hospital income and doctor performance (Table 3). We conducted a topic selection group meeting for doctors at all levels, based on the five main problems identified by coding, summary, and analysis. As there were only five reasons, a score of 5 was assigned for the most important and a score of 1 for the least important reasons. The results showed that the director, deputy director, attending, and resident groups answered that the reasons for improving neuroimaging were the same, in the following order: excluding the risk of misdiagnosis, reducing the uncertainty of diagnosis, avoiding medical disputes, meeting the medical needs of patients, and completing the hospital assessment indicators (Table 4).

**TABLE 3:** Reasons for suggesting neuroimaging to patients with primary headache.

Reason	Example quotation
Excluding the risk of misdiagnosis	“Some headache symptoms are not typical, such as subarachnoid hemorrhage, which may sometimes not be a burst. I will do that because I am afraid of misdiagnosis and missed diagnosis.”
	“The prevalence of headaches is high, and many times you have to be assured. After all, the longer the clinical work time, there will be more and more atypical extreme cases ….”
	“If I have never done a head examination, I can do one, because my eyes are not fluoroscopic, and I am still worried about misdiagnosis and missed diagnosis…”
Reducing the uncertainty of doctors	“Although primary headache is probably considered, there are too many uncertainties in clinical work, and only perfecting the neuroimaging can ensure that patient is safe”
	“…Or to eliminate organic lesions, do a safety point ….”
	“If patients have never done the inspection, I will do it to make me feel safe.”
Avoiding medical disputes	“If the diagnosis is wrong, patients will find a way to complain about you.”
	“It is necessary to do neuroimaging to exclude secondary factors. If you miss the diagnosis, the patient and his family members will complain about you, causing medical disputes.”
	“How can you be 100% sure that there is no other reason; therefore, the way the doctor bears the risk is good; in the medical environment, any misdiagnosis, missed diagnosis, the patient will take you to the end, and the lawsuit will really work.”
Meeting the medical needs of patients	“… But if the patient is desperate to undergo the examination, it is also possible that the test results can relieve the patient's anxiety.”
	“Sometimes inform the patient that he cannot do it and need follow‐up observation; however, the patient's health needs to improve.”
Completing the hospital assessment indicators	“The hospital also has index assessment, how many tests to perform.”
	“…. Also, if the hospital income is high, the income of doctors will be higher.”

**TABLE 4:** Ranking of the reasons of each group after topic interview by scores.

	Excluding the risk of misdiagnosis	Reducing the uncertainty of doctors	Avoiding medical disputes	Meeting the medical needs of patients	Completing the hospital assessment indicators
Four resident clinicians	19	15	9	9	8
Four attending clinicians	19	15	14	7	5
Four associate chief clinicians	19	15	13	7	6
Three chief clinicians	14	12	10	4	5

#### 3.2.2 Is neuroimaging for patients with primary headaches a low‐value intervention?

Three clinicians (two residents and one chief clinician) stated that neuroimaging of patients with primary headaches is low‐value care; they stated that performing neuroimaging for all patients with primary headaches with normal neuroimaging results had wasted medical resources and increased the patients’ medical burden as well as risk of exposure to unnecessary radiation. However, 13 doctors stated that patients with primary headaches who underwent neuroimaging did not receive low‐value care. The reasons for the doctors’ considerations are essentially the same: ensuring medical safety, eliminating the risk of misdiagnosis, avoiding medical disputes, and meeting patients’ medical needs.

#### 3.2.3 Recommendations for reducing low‐value care

Four clinicians made the following suggestions during the interviews: (1) The current follow‐up system is not perfect, and hospitals should improve the outpatient follow‐up rate and shorten the follow‐up time; (2) establish and improve mechanisms for sharing risks between doctors and patients; (3) monitor the medical management system, evaluate doctors with corresponding indicators, and implement performance penalties according to the number of corresponding major examinations; and (4) teach doctors the concept of valuing medical treatment.

## 4 DISCUSSION

Our study found that the neuroimaging rate in patients with primary headaches was very high, occurring in 79.79% of outpatients and 98.13% of inpatients. By conducting topic‐selection group interviews with doctors at all levels, we found that the top concerns for doctors at all levels were misdiagnoses and missed diagnoses.

A previous study revealed that the probability of neuroimaging increased from approximately 5% in 1995 to 15% in 2010 (Callaghan et al., 2014). Similarly, this study found that the probability of neuroimaging in patients with primary headaches was as high as 89.75%. Despite the advocacy of the Choosing Wisely campaign against imaging in patients with primary or typical migraine headaches, this practice remains prevalent, with estimates ranging from 12.4% to 15.9% of patients with primary headaches undergoing MRI in outpatient practice (Elhabr et al., 2022; Jordan et al., 2009). Young et al. reported in 2018 that “an estimated 35% of patients were imaged against guidelines” in their study regarding outpatients with headaches (Callaghan et al., 2015; Gadde et al., 2019). Other studies have demonstrated that up to 31% of patients with headaches require neuroimaging (Young et al., 2018). The reasons for China's higher rate of neuroimaging may be as follows: (1) China is a socialist market economy lacking state investment in health services, and hospitals no longer existing as public welfare institutions struggle for both survival and development and lack adequate income; (2) with the rapid development of medical and treatment instruments and progress in imaging diagnostic technology, some doctors overly rely on various advanced auxiliary diagnostic equipment; (3) owing to patients’ increased awareness of self‐protection, factors such as high medical compensation, and doctors’ desire to avoid subjective mistakes and be free from medical disputes, they use “defensive” medical measures; (4) the lack of medical knowledge—some patients have little knowledge of the disease, believing that the more expensive the examination, the more helpful the diagnosis, and some patients can pay for expensive tests, so that they ask for CT scans or MRI; and (5) China has a large population and a large daily outpatient volume, and there is not enough time to give patients a careful medical and physical examination (Gilbert et al., 2012; Haichao et al., 2002; Yanfeng et al., 2005).

Our study found that neuroimaging requests were unrelated to the professional title, and all interviewed teams were afraid of misdiagnoses, missed diagnoses, safe points (defensive medical treatment), and entanglement. These were the top three reasons for the neuroimaging requests. Consistent with our study, previous studies found that even if no red flag or focal neurological abnormalities existed, doctors in the United States might offer a range of reasons, such as defensive medicine, community standard of care, concerns about professional reputation being affected, fear of litigation and sanctions, avoidance of patient dissatisfaction (such as patients insisting on perfect imaging, especially in pediatrics), and interest in proving that patient imaging is the right choice (Bishop et al., 2017; Howard, 2005; Kerr et al., 2017; Kuruvilla & Lipton, 2015; Quanliang et al., 2006; Rothberg et al., 2014; Scott, 2017; Scott & Elshaug, 2013; Sempere et al., 2005). In addition, the impact of social media, patient dissatisfaction, and satisfaction surveys increase the sensitivity of clinicians to patient preferences and may also cause doctors to struggle to resist strong patient demands (Jordan & Flanders, 2020).

Most clinicians believe that improving imaging for patients with primary headaches is not low‐value care, as previously mentioned, and imaging tests with negative findings may reduce patient or family anxiety, providing an anxiolytic effect for multiple parties (including providers) (Jordan & Flanders, 2020). Other studies have found that other benefits of negative results include improved patient productivity, fewer late visits, fewer psychological and behavioral problems, incidental findings such as sinus disease (the cause of headache), and early treatment (Hermer & Brody, 2010; Jordan et al., 2000; Kennis et al., 2013).

In this study, doctors suggested improving the outpatient follow‐up mechanism and doctor–patient risk‐sharing mechanism, increasing the specific low‐value medical assessment indicators, and increasing the medical value of doctors’ education. Countries have made many efforts to reduce low‐value healthcare services, such as encouraging clinical personnel to provide more valuable treatment or examination using quality‐based compensation, approving low‐value medical treatment only with prior authorization, providing clinicians with more detailed patient information to facilitate clinical decision‐making, providing empirical and cost‐conscious education, supporting clinicians in putting forward suggestions on low‐value medical treatment, implementing supply management techniques (Colla, 2014; Colla et al., 2017; Howard, 2005; Middleton, 2018), and disclosing the quality of treatment to reduce low‐value care (Mafi & Parchman, 2018; Morden et al., 2014). Patient cost‐sharing is the main mechanism for reducing low‐value utilization at the patient level. Patient education partly promotes competition through decision sharing, disclosing the low‐value utilization of medical providers, and providing better demand standards on what to choose (Wise, 2017).

Our study has some limitations. As the treatment information of many patients has not been interconnected, it cannot be directly extracted from the unified system, and the workload of checking each case is prohibitive. Therefore, we only investigated seven hospitals. As we only obtained data from the case system, there was no way to obtain medical insurance and cost‐related information. In the future, we will investigate the influence of insurance in future work.

## 5 CONCLUSIONS

For primary headaches, the probability of performing neuroimaging in China is much higher than in other countries. Doctors in China are more inclined toward performing neuroimaging because most doctors think this is not an excessive treatment. Measures against low‐value medicine and China's participation in Choosing Wisely campaigns should be prioritized.

## AUTHOR CONTRIBUTIONS

*Conceptualization; writing—original draft; writing—review and editing*: X. M. Zhong. *Methodology; data curation*: L. C. Zhao. *Methodology; software*: L. L. Peng. *Data curation; methodology*: L. Li. *Writing—review and editing; supervision; validation; conceptualization; methodology*: C. Q. Li.

## CONFLICT OF INTEREST STATEMENT

The authors declare no conflicts of interest.

## FUNDING INFORMATION

No funding.

### PEER REVIEW

The peer review history for this article is available at https://publons.com/publon/10.1002/brb3.3583.

## Data Availability

The dataset generated for this study can be obtained upon request from the corresponding author.
